# *Pinus densiflora* Root Extract Attenuates Osteoarthritis Progression by Inhibiting Inflammation and Cartilage Degradation in Interleukin-1β and Monosodium Iodoacetate-Induced Osteoarthritis Models

**DOI:** 10.3390/nu16223882

**Published:** 2024-11-14

**Authors:** Young Mi Park, Dong Yeop Shin, Hak Yong Lee, Hai Min Hwang, Jae Gon Kim, Byeong Soo Kim, Sang Ho Lee, Sang Choon Lee, Min Jung Kim, Hye Jeong Yang, Myung-Sunny Kim, Jun Sang Bae

**Affiliations:** 1 INVIVO Co., Ltd., 121, Deahak-ro, Nonsan 32992, Chungnam, Republic of Korea; pym07130@hanmail.net (Y.M.P.); sdy1325@hanmail.net (D.Y.S.); leeapf@nate.com (H.Y.L.); purity464@naver.com (H.M.H.); newstyle.kim@gmail.com (J.G.K.); 2 Department of Pathology, College of Korean Medicine, Wonkwang University, 460, Iksan 54538, Jeonbuk, Republic of Korea; 3 Department of Companion and Laboratory Animal Science, Kongju National University, 54-3 Deahak-ro, Esan-Eub, Yesan-gun 32439, Chungnam, Republic of Korea; bskim@kongju.ac.kr; 4 Sigolsori Farming Association Corporation, 153, Jangpa-gil, Gui-myeon, Wanju-gun 55363, Jeonbuk, Republic of Korea; 2328900@naver.com (S.H.L.); lsc@naver.com (S.C.L.); 5 Korea Food Research Institute, 245, Nongsaengmyeong-ro, Iseo, Wanju-gun 55365, Jeonbuk, Republic of Korea; kmj@kfri.re.kr (M.J.K.); yhj@kfri.re.kr (H.J.Y.); truka@kfri.re.kr (M.-S.K.)

**Keywords:** *Pinus densiflora* root, osteoarthritis, anti-inflammation, cartilage degradation, MAPKs

## Abstract

Background: Osteoarthritis (OA) is a common degenerative joint condition caused by an imbalance between cartilage synthesis and degradation, which disrupts joint homeostasis. This study investigated the anti-inflammatory and joint-improving effects of *Pinus densiflora* root extract powder (PDREP) in both in vitro and in vivo OA models. Methods/Results: In an in vitro OA model, in which SW1353 human chondrosarcoma cells were treated with interleukin (IL)-1β, PDREP treatment significantly reduced the mRNA levels of matrix metalloproteinase (MMP)-1, MMP-3, and MMP-13 while enhancing collagen type II alpha 1 (Col2a1) mRNA level, and decreased IL-6 and prostaglandin E2 (PGE2) levels. In addition, PDREP inhibited the phosphorylation of extracellular signal-regulated kinases (ERK), c-Jun N-terminal kinase (JNK), p38, nuclear factor-kappa B (NF-κB), and the expression of inducible nitric oxide synthase (iNOS). In a monosodium iodoacetate (MIA)-induced OA rat model, the administration of PDREP resulted in decreased OA clinical indices, improved weight-bearing indices and gait patterns, reduced histological damage, and lowered serum inflammatory cytokine and MMPs expression. Furthermore, PDREP downregulated the phosphorylation of ERK, JNK, p38, and NF-κB, as well as the expression of iNOS, consistent with the in vitro findings. Conclusions: These results suggest that PDREP exhibits anti-inflammatory and joint-improving effects and has potential as a therapeutic strategy or functional food for the treatment of OA.

## 1. Introduction

Osteoarthritis (OA) is the most common degenerative joint disease that results from the disruption of homeostasis due to a physiological imbalance in cartilage synthesis and degradation [1,2,3]. Common clinical features include inflammation, pain, joint dysfunction, and deformity, causing mobility impairment, which reduces the quality of life. OA is associated with various risk factors, such as age, joint injury, genetics, and obesity. It is classified as an inflammatory condition characterized by joint inflammation [3,4,5,6].

Many studies have suggested that proinflammatory cytokines and mediators play an important role in the onset and progression of OA [7,8,9,10]. As indicators of OA, the levels of inflammatory mediators, such as tumor necrosis factor-alpha (TNF-α), interleukin (IL)-1β, IL-6, nuclear factor kappa B (NF-κB), and cyclooxygenase 2 (COX-2) increase in cartilage and synovial tissues, as well as in synovial fluid [11,12,13]. Additionally, OA is positively correlated with the expression of matrix metalloproteinases (MMPs). MMPs play a crucial role in cartilage matrix degradation in OA, contributing to cartilage tissue degeneration and damage of cartilage tissue [3,11,14]. Notably, MMP-1, MMP-3, and MMP-13 were found to have increased expression in OA and have been reported to be closely linked to OA development [15,16,17,18]. Despite various studies, the optimal treatment method for OA remains unclear [19,20]. General therapeutic approaches for OA aim to regulate inflammation, alleviate pain, and delay or prevent joint damage through long-term pharmacotherapy, physical therapy, surgery, or injection therapy [18,20,21]. However, these treatment methods have side effects, such as gastrointestinal disorders, liver and kidney dysfunction, and inflammation recurrence [21,22]. Therefore, it is crucial to alleviate these side effects and identify new natural products or ingredients with potential protective and anti-inflammatory effects against OA.

*Pinus densiflora*, commonly known as the pine tree, has been cultivated in Korea, Japan, and Northeast China for a long time, with all its parts, including needles, bark, cones, pollen, and roots, being utilized as a valuable herbal medicine [23,24]. In the Orient, *P. densiflora* is mainly used to treat stroke, gastrointestinal disease, atherosclerosis, hypertension, diabetes, and neurological diseases [23,24,25]. Current studies on *P. densiflora* focus on pine needles, which are rich in flavonoids, anthocyanins, carotene, and volatile components such as α-pinene, β-pinene, and camphene. Compounds found in pine needles have been shown to have pharmacological effects such as antioxidant, antibacterial, anticancer, and anti-inflammatory effects [24,25]. Although the biological activity of *P. densiflora* needles as a natural product is well known, research on the components of *P. densiflora* roots and their bioactivities is still insufficient.

In a previous study, water-soluble neutral polysaccharides such as amylose and glucomannan were identified from *P. densiflora* root extract [26]. A recent study found that *P. densiflora* root extract contains terpenoids and triterpenoids with antimalarial, antibacterial, and anticancer properties [23]. Additionally, it was confirmed that *P. densiflora* root extract exhibits antioxidant and anti-inflammatory effects [24,27]. Previous studies have reported that marine pine bark extract (Pycnogenol) alleviates OA symptoms [28,29]. Despite these findings, the effects of *P. densiflora* root extract on OA are not clearly known.

The induction of IL-1β and a monosodium iodoacetate (MIA) serve as established in vitro and in vivo models, respectively, to elucidate the underlying mechanisms of OA and assess the anti-osteoarthritic effects of potential therapeutic drugs [30]. Given the reported antioxidant and anti-inflammatory properties of *P. densiflora*, we hypothesized that *P. densiflora* root extract might exhibit joint-protective and anti-inflammatory effects in OA models by potentially modulating inflammatory mediators and cartilage-degrading enzymes. Therefore, in this study, we investigated the anti-inflammatory and joint protective effects using IL-1β-induced in vitro and MIA-induced in vivo OA models.

## 2. Materials and Methods

### 2.1. Samples

*Pinus densiflora* root extract powder (PDREP) was provided by the Sigolsori, Farming Association Corporation (Wanju, Jeonbuk, Republic of Korea). The sample was diluted to a concentration of 8 mg/mL using distilled water. The diluted sample was centrifuged (12,000 rpm, 4 °C, 10 min) to collect only the supernatant, filtered through a 0.22 μm syringe filter (Pall Co., Port Washington, NY, USA), and analyzed by UPLC–QTOF–MS (Ultra-performance liquid chromatography–quadrupole time of flight mass spectrometer; Synapt G2-Si, Waters Co., Milford, MA, USA). Methyl sulfonyl methane (MSM) was purchased from Mirae Biotech Co., Ltd. (Gyeonggi, Republic of Korea). The MSM, which is used as an OA supplement, was used as a positive control.

### 2.2. UPL–-QTO–-MS Analysis and Data Processing

To obtain a chromatogram of the metabolite profile for PDREP, UPLC–QTOF–MS (Waters Co.) equipped with an ACQUITY^®^ BEH C18 column (2.1 × 100 mm, 1.7 µm; Waters Co.) was used. The mobile phase consisted of (A) 0.1% formic acid in H_2_O and (B) 0.1% formic acid in acetonitrile for chromatographic separation. ESI ions were performed in negative ion mode, and the mass range is 50–1200 *m*/*z*. MS^E^ mode was used as a condition for CID (collision-induced dissociation). To ensure accuracy, sodium formate was used as a calibration material before sample injection, and the detected mass value was corrected using leucine enkephalin (negative 554.2615 *m*/*z*) as an internal reference material (Lockmass; Waters Co.). Detailed analytical conditions are as follows: Column temperature: 35 °C, Injection volume: 5 µL, Flow rate: 0.40 mL/min, Capillary voltage: 2.3 kV, Sample cone: 40 V, Source temperature: 110 °C, Desolvation temperature: 350 °C, Desolvation Gas Flow: 800 L/h, Collision energy ramping: 25–50 eV.

Chromatogram and mass spectrometry data obtained through UPLC–QTOF–MS were standardized using Progenesis QI (Waters Co.) software version 3.0. All data collected through TOF-MS went through the process of chromatographic peak alignment, peak picking, normalization, and compound identification to obtain molecular formula candidates within retention time and tolerance of 10 ppm, and to calculate accurate mass values and intensities of each peak. Flavonoid individual components were identified by referring to ChemSpider (www.chemspider.com (accessed on 24 October 2023)), METLIN database (metlin.scripps.edu (accessed on 24 October 2023)), in-house, and references related to flavonoid glycosides of previously reported materials. The analysis of the metabolite components of PDREP using UPLC–QTOF–MS is presented in Supplementary Figure S1 and Table S1.

### 2.3. Cell Culture and Cell Viability Assay

SW1353 chondrosarcoma cells were purchased from the American Type Culture Collection (ATCC, Rockville, MD, USA) and grown in Dulbecco’s modified Eagle’s medium (DMEM) (Invitrogen, Carlsbad, CA, USA) containing 10% fetal bovine serum (FBS) (Gibco BRL, Gaithersburg, MD, USA) and 1% antibiotic-antimycotic (Invitrogen). The cells were maintained in a humidified incubator at 37 °C with 5% CO_2_ and subcultured every 2–3 days. To analyze the cell viability rate, SW1353 cells (5 × 10^3^ cells/well) were seeded in a 96-well culture plate, treated with varying concentrations of PDREP or MSM, and cultured at 37 °C and 5% CO_2_ for 24 h. After 24 h, 10 µL of water-soluble tetrazolium salt-1 (WST-1) solution (DoGENBio, Seoul, Republic of Korea) was added to 100 µL of cell culture medium, incubated for 1 h, and the absorbance at 450 nm was measured using a Multi Detection Reader (Infinite 200, TECAN Group Ltd., Männedorf, Switzerland). The control group was designated as the experimental group, in which the sample remained untreated, and only the solvent in which the sample was dissolved underwent treatment at the same concentration as the high-concentration experimental group. Cell viability assays were performed for at least three independent experiments (n = 3).

### 2.4. Quantitative Reverse Transcriptase-Polymerase Chain Reaction (qRT-PCR) Analysis

SW1353 cells were distributed into 6-well plates at 3 × 10^5^ cells/mL, PDREP (0, 30, 100, or 300 µg/mL) or MSM (300 µg/mL) was pretreated and then stimulated with IL-1β (0.5 ng/mL) for 24 h. Total RNA was extracted using Trizol reagent (Sigma-Aldrich, St. Louis, MO, USA) and quantified using Quibit 4 reagent calculator (Invitrogen). cDNA was synthesized using 1 µg of quantified total RNA as a template using the Primescript RT reagent kit (Takara Bio, Shiga, Japan). Real-time PCR was performed using Power SYBR Green RT-PCR Master Mix Kit (Applied Biosystems, Foster City, CA, USA). Each reaction mixture consisted of 1 µL of cDNA, 1.5 µL of each primer set (10 pmol), 25 µL of 2× Power SYBR Green RT-PCR master mix, and 21 µL of RNase-free water, for a total of 50 µL. The reaction solution was amplified according to the conditions using QuantStudio™ 1 Real-Time PCR System (Applied Biosystems), the results were corrected for the expression level of the housekeeping gene (GAPDH), and the 2^−∆∆Ct^ result was analyzed. At least three independent experiments were performed (n = 3). The primer sequences used for qRT-PCR are listed in Table 1.

### 2.5. Measurement of NO and PGE2 Production

To measure nitric oxide (NO) and prostaglandin E2 (PGE2) production, SW1353 cells were seeded in 48-well plates at a concentration of 2 × 10^4^ cells/400 µL/well and cultured for 24 h. Cells were added with PDREP (0, 30, 100, or 300 µg/mL) or MSM (300 µg/mL), then treated with IL-1β (0.5 ng/mL) and cultured for 24 h. After incubation, the culture supernatant was collected, and the amounts of NO and PGE2 were measured using ELISA kits (NO; Abcam, Cambridge, UK; PGE2; Mybiosource, San Diego, CA, USA) according to the manufacturer’s protocols. At least three independent experiments were performed (n = 3).

### 2.6. Western Blot Analysis

SW1353 cells (3 × 10^5^ cells/mL) were seeded on a 100 mm dish. The samples were pretreated and then incubated with IL-1β (10 ng/mL) for 24 h. After incubation, the cells were washed three times with cold phosphate-buffered saline (PBS) and lysed using PRO-PREP^TM^ protein extraction solution (iNtRON, Seongnam, Republic of Korea). Briefly, in the animal study, tissue samples were extracted from the left knee joint cartilage of the animals, and protein extraction solution (iNtRON) was added. The collected cells and tissues were disrupted using a vortex and a homogenizer, respectively, followed by centrifugation at 14,000 rpm for 10 min at 4 °C. The extracted protein was quantified using Bradford reagent (Bio-Rad, Hercules, CA, USA). Electrophoresis was performed using a sodium dodecyl sulfate-polyacrylamide gel electrophoresis (SDS-PAGE) system, and the proteins were transferred onto a polyvinylidene fluoride (PVDF) membrane. The membrane was then blocked with a 5% skim milk solution for 1 h. The primary antibody was added and incubated overnight at 4 °C, followed by the addition of a secondary antibody containing horseradish peroxidase (HRP) for 1 h. The membrane was washed with PBS-Tween 20, treated with ECL solution (EZ-Western Lumi Pico, DoGen, Republic of Korea), and detected using a C-Digit Western scanner (LI-COR, Lincoln, NE, USA). The following primary antibodies were used in the experiments: Primary antibodies against ERK, p-ERK, JNK, p-JNK, p38, p-p38, NF-kB, and p-NF-kB were purchased from Cell Signaling Technology (Danvers, MA, USA). The anti-iNOS antibody was purchased from Abcam. The anti-β-actin antibody was purchased from Santa Cruz Biotechnology (Dallas, TX, USA). The catalog numbers and dilution ratios of the antibodies used are listed in Supplementary Table S2.

### 2.7. Animals and Experimental Design

The experimental animals were 8-week-old male Sprague-Dawley (SD) rats, which were purchased from Samtako Bio Korea (Osan, Republic of Korea) and used in the experiment after a one-week acclimatization period. During the adapting period, the temperature was maintained at 23 ± 1 °C, humidity at 50 ± 5%, noise levels less than 60 phons, lighting for 12 h a day from 07:00 to 19:00, illuminance ranging from 150 to 300 lx, and ventilation occurring 10–12 times per hour. All the animals were subjected to veterinary quarantine to assess their overall health. The animals were provided with a standard rodent diet (Samtako) and allowed free access to sterilized water. After the adaptation period, the SD rats were weighed, and the mean values were equally divided into six groups (10 rats per group) according to a randomized block design: normal group (Normal), MIA-induced arthritis model group (Control), MIA + PDREP treatment group (PDREP 100 mg/kg; PDREP 300 mg/kg; PDREP 500 mg/kg), and MIA + MSM treatment group (MSM 300 mg/kg, positive control).

PDREP (100, 300, and 500 mg/kg) and MSM (300 mg/kg) were orally administered for six weeks. In the 3rd week of sample administration, MIA was injected to induce arthritis. For the MIA-induced arthritis model, rats were anesthetized with isoflurane, and 50 µL of MIA (60 mg/mL) diluted with 0.9% sodium chloride was injected into the joint cavity of the left knee using an insulin syringe. The experimental protocol scheme and body weight changes following PDREP treatment in the MIA-induced OA rat model are shown in Supplementary Figure S2. This study was approved by the Institutional Animal Care and Use Committee of INVIVO Co., Ltd. (approval no. IV-RB-02-2304-09).

### 2.8. Arthritis Clinical Index, Weight-Bearing Index and Gait Analysis

The arthritis clinical index was independently assessed by four researchers, as previously described [31]. The degree of knee joint swelling and bending in each experimental group was assessed and graded on a scale of 0–3 points based on severity. The clinical index of arthritis was scored as 0 (no change), 1 (mild swelling of the ankle joint), 2 (moderate swelling of the ankle joint through the metatarsal bone), or 3 (severe swelling of the ankle joint through the digits). Scores were assigned, and the results were reported as average values. The hind limb weight-bearing assessment was performed within a controlled plastic holder set at a 60-degree inclination using an incapacitance meter tester (IMT). Subsequently, the strength exerted on each hind limb was measured and averaged over 5 s. The body weight distribution over the treated ipsilateral hind limbs was calculated using the following formula:

Weight-bearing index (%) = (weight of induced lower extremity/weight of normal lower extremity) × 100.

For the gait analysis, hind paw prints of the experimental animals were obtained by applying ink to the hind paws. The experimental animals were allowed to run on white paper 60 cm in length and 7 cm in width, and the paw area and width were quantified using ImageJ software version 1.8.0 (NIH, Bethesda, MD, USA).

### 2.9. Serum Biochemical Parameter Analysis

After the final administration of all drugs and vehicles, blood samples were obtained from the abdominal vena cava of the animals following inhalation anesthesia and transferred to conical tubes for analysis. For serum biochemical parameter analysis, the blood collected in conical tubes was allowed to coagulate for 30 min at room temperature. Serum was recovered by centrifugation at 3000 rpm for 10 min. The separated serum was measured for the contents of TNF-α (Cusabio, Houston, TX, USA), IL-6 (Mybiosource), PGE2 (Cusabio), MMP-1 (Mybiosource), MMP-3 (Mybiosource), MMP-13 (Mybiosource), and NO (Invitrogen) using an ELISA kit according to the manufacturer’s instructions.

### 2.10. Micro-Computed Tomography (Micro-CT) Analysis

Micro-CT measurement was performed using a Bruker SkyScan 1173 (Bruker, Kontich, Belgium) under the following parameters: voltage of 90 kVp, current of 88 µA, a pixel size of 15 µm, rotation step (deg.) of 0.3, angular step (deg.) of 0.3, and an exposure of 500 ms. Image analysis was performed using SkyScan 1173 (Bruker, Kontich, Belgium). For this analysis, five animals per group were selected after the experiment was completed, volume rendering was performed, and 3D images were acquired.

### 2.11. Histological Analysis

After completion of the experiment, each animal underwent an autopsy, and the left knee joint was excised for histopathological examination. Resected knee joints were fixed in 10% neutral buffered formalin (Sigma-Aldrich) for 24 h, followed by decalcification in 10% EDTA for 20 h, then embedded in paraffin, and sectioned into 5 µm-thick slices. To assess histological findings, paraffin-embedded tissue sections were stained with hematoxylin and eosin (H&E) and Safranin-O. All specimens were examined by pathology experts using an EasyScan slide scanner (Motic, Hong Kong, China), and blind testing was conducted to exclude subjective judgment. The stained tissues were analyzed to grade osteoarthritic damage using the Osteoarthritis Research Society International (OARSI) Scoring System [32].

### 2.12. Statistical Analysis

The cell and animal experimental results were calculated as mean ± standard deviation (mean ± S.D.) and mean ± standard error (mean ± S.E.), respectively, using the statistical program SPSS version 23.0 (SPSS Inc., Chicago, IL, USA). Statistical analyses, including one-way analysis of variance (ANOVA), were performed to test for significance between the experimental groups. If significance was observed, post-hoc testing was conducted using Duncan’s multiple range test at a significance level of *p* < 0.05.

## 3. Results

### 3.1. Effect of PDREP on Cell Viability and Inflammatory Mediator Production in SW1353 Cells

**Figure 1:**
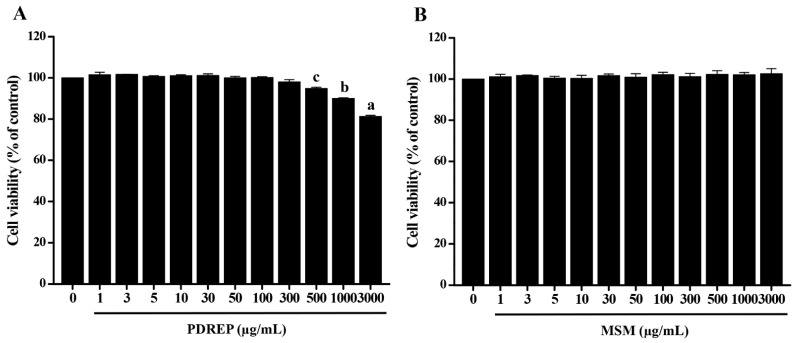
Effect of PDREP on the viability of SW1353 cells. SW1353 cells (5 × 10^3^ cells/well) were seeded in 96-well plates and treated with (**A**) PDREP or (**B**) MSM at concentrations ranging from 1 to 3000 μg/mL, respectively. The cells were then incubated at 37 °C with 5% CO_2_ for 24 h. After incubation, cell viability was measured using the WST-1 assay. Bars labeled with different superscript letters indicate significant differences (*p* < 0.05 vs. control). The results are expressed as mean ± SD of at least three independent experiments (n = 3).

To confirm the effect of PDREP on OA, cytotoxic concentrations of PDREP were tested. The SW1353 cells were treated with different concentrations of PDREP for 24 h. As a result of measuring the cell viability after 24 h, the cell viability was more than 80% from 1 µg/mL of PDREP to the highest dose of 3000 µg/mL of PDREP. As PDREP concentrations of 1 to 300 µg/mL showed a cell survival rate of over 95%, subsequent experiments were conducted with the PDREP sample treatment concentration set to a maximum of 300 µg/mL (Figure 1A). In the case of MSM used as a positive control, it was shown to be non-cytotoxic at all sample concentrations, so subsequent experiments were conducted at the same concentration of 300 µg/mL, the highest concentration of the PDREP sample (Figure 1B).

**Figure 2:**
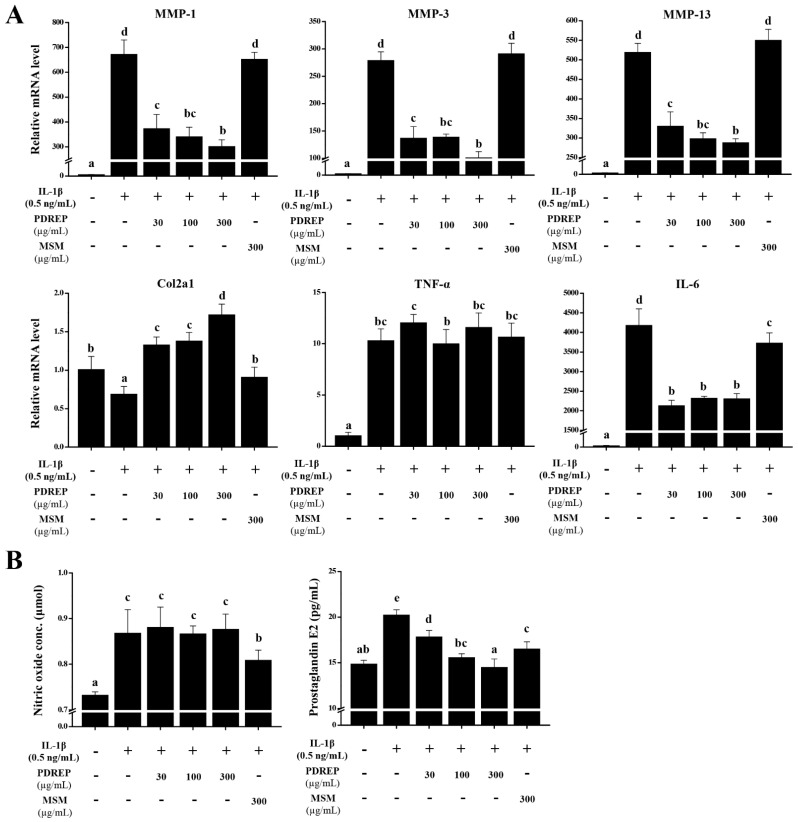
Effect of PDREP on the expression of MMPs and inflammatory cytokine-related genes, and NO and PGE2 production in SW1353 cells. (**A**) SW1353 cells (3 × 10^5^ cells/well) were seeded in 6-well plates, treated with PDREP (0, 30, 100, or 300 µg/mL) or MSM (300 µg/mL), and then stimulated with IL-1β (0.5 ng/mL) for 24 h. The expression of genes (*MMP1*, *MMP3*, *MMP13*, *Col2a1*, *TNF-α*, and *IL6*) was evaluated by qRT-PCR. (**B**) SW1353 cells (2 × 10^4^ cells/well) were dispensed into a 48-well plate. The cells were then treated with PDREP (0, 30, 100, or 300 µg/mL) or MSM (300 µg/mL), followed by treatment with IL-1β (0.5 ng/mL), and cultured for 24 h. The levels of NO and PGE2 in the culture supernatant after incubation were quantified using ELISA kits following the manufacturer’s protocol. The experiments were conducted in triplicate, and the error bars represent the standard deviation. Significant differences between the PDREP and control groups are indicated (*p* < 0.05). Bars labeled with different superscript letters indicate *p* values < 0.05.

To determine whether PDREP affects the expression of MMPs, inflammatory cytokines, and mediators that cause the destruction of articular cartilage tissue and inflammation, were analyzed using qRT-PCR or ELISA. After PDREP pretreatment, IL-1β was added and cultured for 24 h, and the mRNA levels of MMP-1, MMP-3, MMP-13, Col2a1, TNF-α, and IL-6 were confirmed. As a result, the mRNA level of MMP-1, MMP-3, MMP-13, TNF-α, and IL-6 significantly increased in the IL-1β alone treatment group compared to the untreated group, but the mRNA level of Col2a1 significantly decreased. Interestingly, MMP-1, MMP-3, and MMP-13 levels elevated by IL-1β were significantly decreased by PDREP in a dose-dependent manner. On the contrary, the Col2a1 levels reduced by IL-1β exhibited a dose-dependent significant increase in the PDREP groups. Additionally, the IL-6 levels elevated by IL-1β exhibited a significant decrease in the PDREP groups ranging from 30 to 300 µg/mL, but there was no significant difference in the mRNA level of TNF-α. When comparing the MSM treatment group with the IL-1β treatment group alone, there was no significant difference in the mRNA levels of MMP-1, MMP-3, MMP-13, and TNF-α, excluding Col2a1 and IL-6 (Figure 2A). Finally, to confirm the effect of PDREP on NO and PGE2 content increased by IL-1β, they were analyzed using an ELISA kit. As a result of the PGE2, the elevated levels of PGE2 induced by IL-1β indicated a dose-dependent, significant reduction in the PDREP groups. In particular, at the highest concentration of PDREP, 300 µg/mL, it was significantly suppressed to the level of the untreated group. However, the NO production elevated by IL-1β showed no significant difference in the PDREP groups. When comparing the MSM treatment group with the IL-1β treatment group alone, the MSM treatment group showed a significantly decrease in PGE2 and NO levels (Figure 2B).

### 3.2. Effect of PDREP on the MAPKs and NF-κB Activation in SW1353 Cells

**Figure 3:**
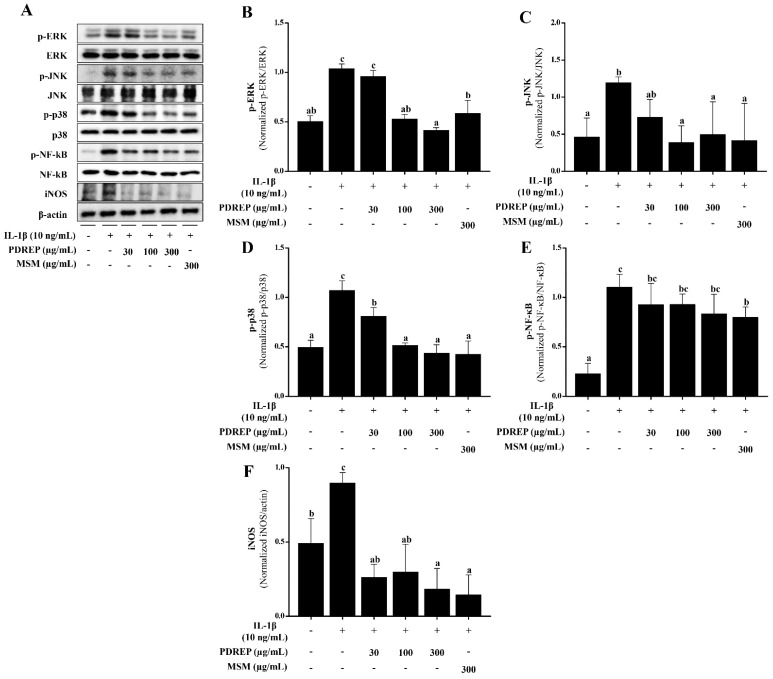
Effect of PDREP on MAPKs and NF-κB activation in SW1353 cells. SW1353 cells were cultured with PDREP (0, 30, 100, or 300 µg/mL) or MSM (300 µg/mL) and then treated with IL-1β (10 ng/mL) for 24 h. (**A**) Protein expression levels were analyzed by Western blotting using anti-ERK, p-ERK, p38, p-p38, JNK, p-JNK, NF-κB, p-NF-κB, and iNOS antibodies. (**B**–**F**) Band intensities were quantified using ImageJ software. Values are presented as mean ± SD (n = 3). Bars labeled with different superscript letters indicate *p* < 0.05.

In this study, it was confirmed that PDREP suppresses inflammatory mediators in the IL-1β-stimulated SW1353 cells. Based on these findings, Western blot analysis was conducted to investigate the effects of PDREP on the MAPKs (ERK, JNK, p38) and NF-κB signaling pathways, which play crucial roles in the inflammatory response in IL-1β-induced SW1353 cells. The phosphorylation levels of MAPKs, NF-κB, and iNOS expression increased in the IL-1β-only treatment group compared to the untreated group. On the other hand, the level of p-ERK increased by IL-1β treatment was significantly decreased in the groups treated with PDREP 100 μg/mL and PDREP 300 μg/mL. In the case of p-JNK, the expression level of p-JNK significantly decreased in all experimental groups compared to the control group. The expression level of p-p38 significantly decreased in a PDREP-dose-dependent manner. The expression level of p-NF-κB tended to decrease in a PDREP concentration-dependent manner. Additionally, the iNOS expression level was found to be significantly decreased compared to the control group treated with IL-1β only at all concentrations of PDREP. In the MSM-treated group, the phosphorylation levels of MAPKs, NF-κB, and iNOS protein levels were significantly decreased compared to the control group treated only with IL-1β (Figure 3A). The expression of each protein was normalized, and the relative expression levels were analyzed (Figure 3B–F).

### 3.3. Effect of PDREP on Arthritis Clinical Index, Weight-Bearing Index, and Gait in MIA-Induced OA Rats

**Figure 4:**
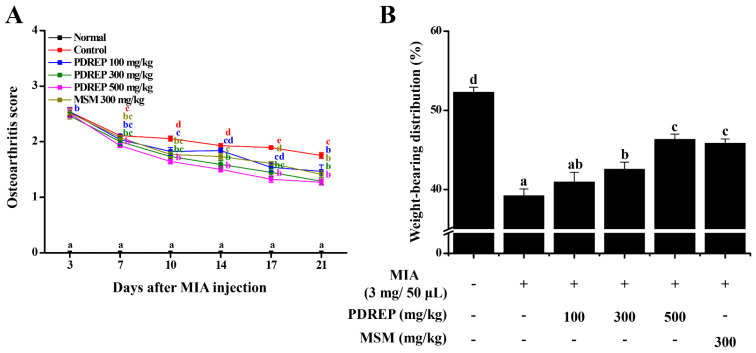
Effect of PDREP on the osteoarthritis score and the change of weight-bearing in MIA-induced OA rats. The 60 SD rats were divided into six groups (10 rats per group) and were orally administered treatments for six weeks: normal control group (Normal), MIA-induced OA group (Control), MIA injection + PDREP 100 mg/kg group (PDREP 100 mg/kg), MIA injection + PDREP 300 mg/kg group (PDREP 300 mg/kg), MIA injection + PDREP 500 mg/kg group (PDREP 500 mg/kg), and MIA injection + MSM 300 mg/kg group (MSM 300 mg/kg). In the 3rd week of sample administration, MIA was injected to induce arthritis. (**A**) Osteoarthritis score was evaluated once every 3 or 4 days. (**B**) The index of weight-bearing (weight of induced lower extremity/weight of normal lower extremity × 100). Bars labeled with different superscript letters indicate *p* values < 0.05. Data are expressed as mean ± SE (n = 10).

In this study, the clinical index of OA was evaluated to confirm the effect of PDREP in an MIA-induced OA rat model. The analysis revealed that the normal group (Normal), in which OA was not induced, showed no specific joint symptoms. However, in the control group (Control), OA was induced by MIA, and severe limping and inability to stand were observed. These symptoms in the control group appeared to decrease over time, but no significant improvement was observed, with a relatively high score of 1.75 ± 0.05 observed on day 21, the time of autopsy. In contrast, in the experimental group administered PDREP or MSM, symptoms appeared to be alleviated over time compared to the control group following MIA administration. The OA clinical index was 1.46 ± 0.11 points for the PDREP 100 mg/kg group, 1.29 ± 0.06 points for the PDREP 300 mg/kg group, and 1.27 ± 0.05 points for the PDREP 500 mg/kg group. The positive control group, treated with MSM 300 mg/kg, expressed a score of 1.41 ± 0.07 points (Figure 4A).

We also analyzed the weight-bearing index and confirmed the pain improvement effect of PDREP in a rat model of MIA-induced OA. As a result, it was confirmed that the average weight-bearing of both hind limbs of the normal group was the same at 52.31 ± 0.62%. In contrast, the weight-bearing index of the control group was 39.21 ± 0.86%, which was significantly reduced compared to the normal group, confirming that MIA administration caused pain. In the OA rat model, weight-bearing index was significantly increased in the PDREP 300 mg/kg group (42.57 ± 0.88%), PDREP 500 mg/kg group (46.33 ± 0.66%), and MSM 300 mg/kg group (positive control, 45.84 ± 0.54%), excluding the PDREP 100 mg/kg group (40.96 ± 1.23%) compared to the control group (Figure 4B).

**Figure 5:**
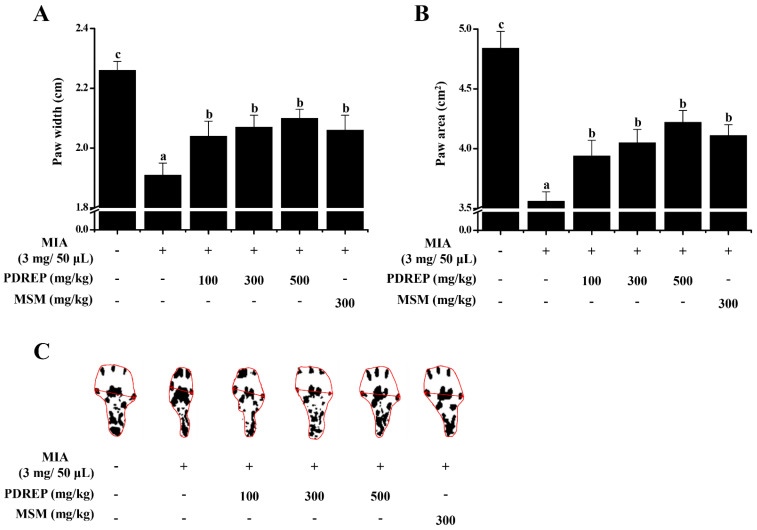
Effect of PDREP on the gait improvement in MIA-induced OA rats. In the MIA-induced OA rat model, we analyzed gait patterns by measuring (**A**) paw width and (**B**) paw area. (**C**) Representative images of paw width and paw area measured on day 21 of the study are presented. Values in the row with different superscript letters are significantly different, *p* < 0.05. Data are expressed as mean ± SE (n = 10).

Additionally, gait analysis was performed as another method to confirm the pain improvement effect of PDREP in the MIA-induced OA rats. The paw width and area of the control group were found to be 1.91 ± 0.04 cm and 3.56 ± 0.08 cm^2^, respectively, which were significantly decreased compared to 2.26 ± 0.03 cm and 4.84 ± 0.14 cm^2^ of the normal group. The paw width and area values of the experimental groups were PDREP 100 mg/kg group (2.04 ± 0.05 cm and 3.94 ± 0.13 cm^2^), PDREP 300 mg/kg group (2.07 ± 0.04 cm and 4.05 ± 0.11 cm^2^), PDREP 500 mg/kg group (2.10 ± 0.03 cm and 4.22 ± 0.10 cm^2^), and MSM 300 mg/kg group (2.06 ± 0.05 cm and 4.11 ± 0.09 cm^2^), respectively. Similar to the weight-bearing test results, foot width and area values significantly increased in all experimental groups, including the positive control group (Figure 5A–C).

### 3.4. Effect of PDREP on Serum Biochemical Parameters in MIA-Induced OA Rat Model

**Figure 6:**
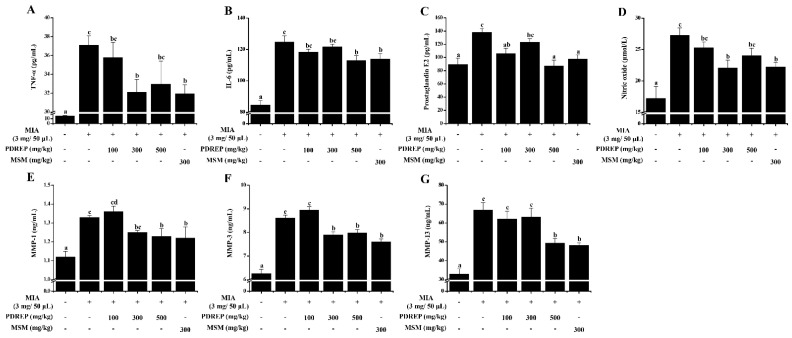
Effect of PDREP on serum biochemical parameters in MIA-induced OA rat model. For serum biochemical parameters analysis, blood collected in a conical tube was coagulated at room temperature for 30 min and then separated in a centrifuge at 3000 rpm for 10 min to collect serum. The separated serum was analyzed for (**A**) TNF-α, (**B**) IL-6, (**C**) PGE2, (**D**) NO, (**E**) MMP-1, (**F**) MMP-3, and (**G**) MMP-13 levels using an ELISA kit. Bars labeled with different superscript letters indicate *p* < 0.05. Data are expressed as mean ± SE (n = 10).

Based on the improvement in clinical symptoms and pain in the OA rat model by PDREP, serum biochemical indicators, including inflammatory cytokines, were analyzed. As a result, it was confirmed that the levels of TNF-α, IL-6, PGE2, and NO were significantly increased in the serum of the MIA-induced control group compared to the normal group. TNF-α content showed a pattern in which the PDREP group had suppressed TNF-α levels compared to the control group. Particularly, the PDREP 300 mg/kg and MSM 300 mg/kg groups showed stronger inhibitory effects (Figure 6A). In the case of IL-6, similar to TNF-α, the experimental groups exhibited a pattern of reducing IL-6 levels compared to the control group, with significantly decreased IL-6 levels noted in the PDREP 500 mg/kg group and MSM 300 mg/kg group (Figure 6B). The MIA-induced increase in PGE2 levels was significantly decreased in all experimental groups except for the PDREP 300 mg/kg group (Figure 6C). In addition, the PDREP treatment groups appeared to show a pattern of decreased NO levels, and the PDREP 300 mg/kg and MSM 300 mg/kg groups showed a significant decrease (Figure 6D). Our results also showed that PDREP reduced the levels of MMP-1, MMP-3, and MMP-13, which increased following MIA treatment. The levels of MMP-1 and MMP-3 were significantly decreased in the PDREP 300 and 500 mg/kg groups compared with those in the control group. In the case of MMP-13, the PDREP 500 mg/kg group showed a significant decrease (Figure 6E–G).

### 3.5. Effect of PDREP on the Knee Joints Cartilage Volume in MIA-Induced OA Rats

**Figure 7:**
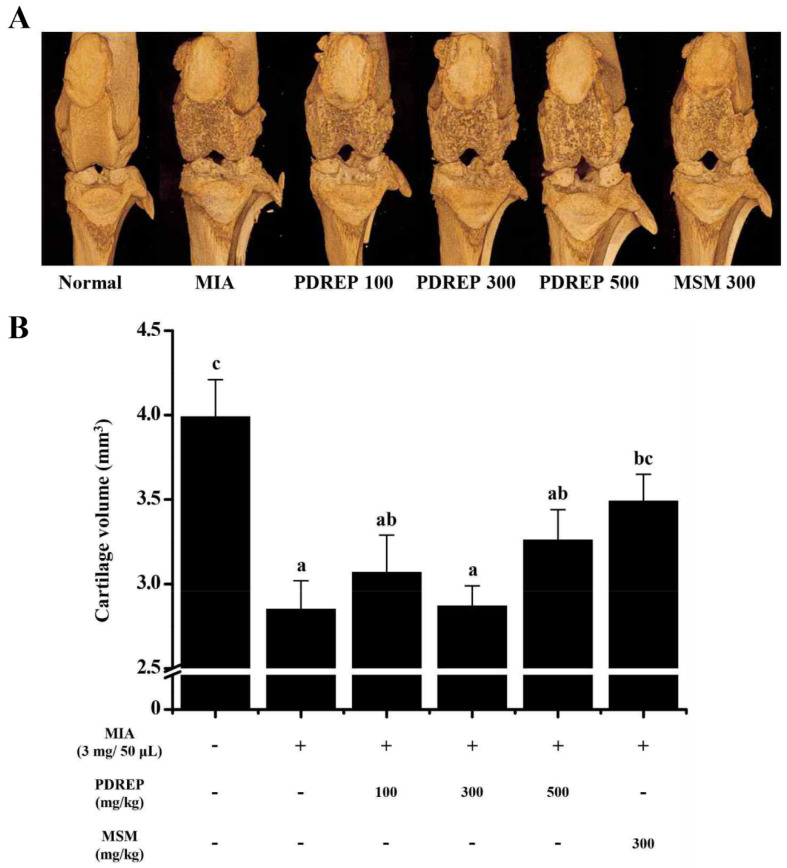
Effects of PDREP on knee joint volume in MIA-induced OA rats. (**A**) Three-dimensional micro-CT images and (**B**) cartilage volume were assessed using the micro-CT system. The data are expressed as the mean ± SE (n = 5), and different letters indicate a significant difference at *p* < 0.05.

Joint cartilage, located between the knee joints, protects against and absorbs shock and supports proper knee function. Therefore, we morphologically evaluated the articular cartilage using micro-CT arthrography to investigate how PDREP affects the structure of the knee joint (Figure 7A). As shown in Figure 7B, it was confirmed that the volume of cartilage was significantly reduced in the control group (2.85 ± 0.17 mm^3^) compared to the normal group (3.99 ± 0.22 mm^3^). The decreased cartilage volume induced by MIA was observed to be preserved when treated with high-dose PDREP 500 mg/kg (3.26 ± 0.18 mm^3^) and MSM 300 mg/kg (3.49 ± 0.16 mm^3^).

### 3.6. Effect of PDREP on Histopathological Features of Knee Joint Tissue in OA Rats

**Figure 8:**
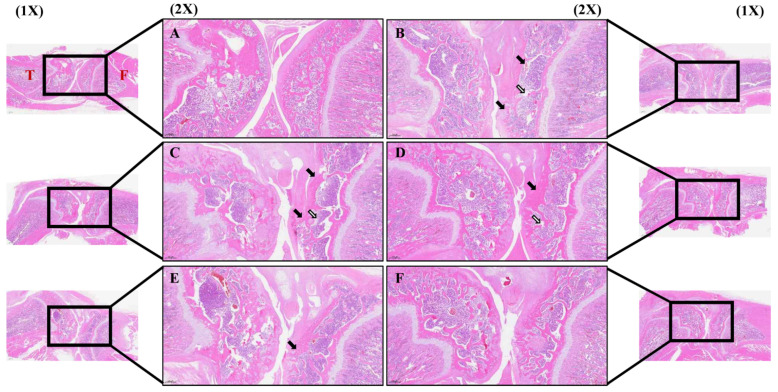
The histological analysis of synovial cell changes and inflammatory cell infiltration in the knee joint tissues after treatment with PDREP in MIA-induced OA rats. Histological changes were assessed using hematoxylin and eosin (H&E) staining: (**A**) Normal, (**B**) Control, (**C**) PDREP 100 mg/kg, (**D**) PDREP 300 mg/kg, (**E**) PDREP 500 mg/kg, (**F**) MSM 300 mg/kg. Histological analysis results image magnification = 1× and 2×, and scale bar = 1000 µm and 500 μm, respectively. Square: High magnification (2×), Black arrows: articular cartilage loss, White arrows: reduced chondrocyte numbers, subchondral bone collapse. T: Tibia, F: Femur.

**Figure 9:**
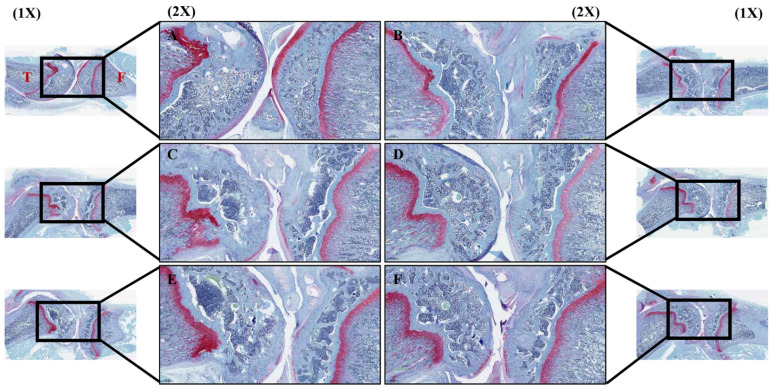
The histological analysis of cartilage tissue damage through Safranin-O staining in MIA-induced OA rats. (**A**) Normal, (**B**) Control, (**C**) PDREP 100 mg/kg, (**D**) PDREP 300 mg/kg, (**E**) PDREP 500 mg/kg, (**F**) MSM 300 mg/kg. Histological analysis results image magnification = 1× and 2×, and scale bar = 1000 µm and 500 μm, respectively. Square: High magnification (2×). T: Tibia, F: Femur.

To investigate whether PDREP altered synovial cells within the knee joint and infiltrated inflammatory cells in the rat model of OA induced by MIA, H&E staining was performed. H&E staining revealed that, in the normal group, synovial tissues exhibited a regular arrangement and no inflammatory cells (Figure 8A). The control group exhibited a significant loss of articular cartilage tissue around the joints, pronounced infiltration of inflammatory cells within the tissue, and significant subchondral bone collapse (Figure 8B). In contrast, it was observed in the experimental group treated with PDREP or MSM that, with increasing administered concentrations, the alteration of cartilage, synovium, and fibrous tissue was mitigated compared to the control group (Figure 8C–F). Additionally, we examined the damage to the cartilage tissue by staining the proteoglycan layer of cartilage cells within the knee cartilage with Safranin-O. As a result, the joints in the normal group showed normal cartilage tissue arrangement, and the proteoglycan layer was strongly stained in red (Figure 9A). In contrast, the control group displayed inflammatory cell infiltration around the joint, leading to the destruction of cartilage tissue and significant disappearance of the proteoglycan layer (Figure 9B). In contrast, there was no clear difference between the PDREP groups, but compared to the control group, the red area was significantly increased in the PDREP and positive control groups (MSM) (Figure 9C–F). Additionally, the OARSI scores revealed significant differences among the groups. OARSI scores were markedly higher in the control group than in the normal group. The PDREP or MSM groups showed a significant decrease in OARSI scores compared to the control group, suggesting a protective effect against cartilage degradation (Supplementary Figure S3).

### 3.7. The Expression Levels of MAPKs and NF-κB Proteins by PDREP in MIA-Induced OA Rats

**Figure 10:**
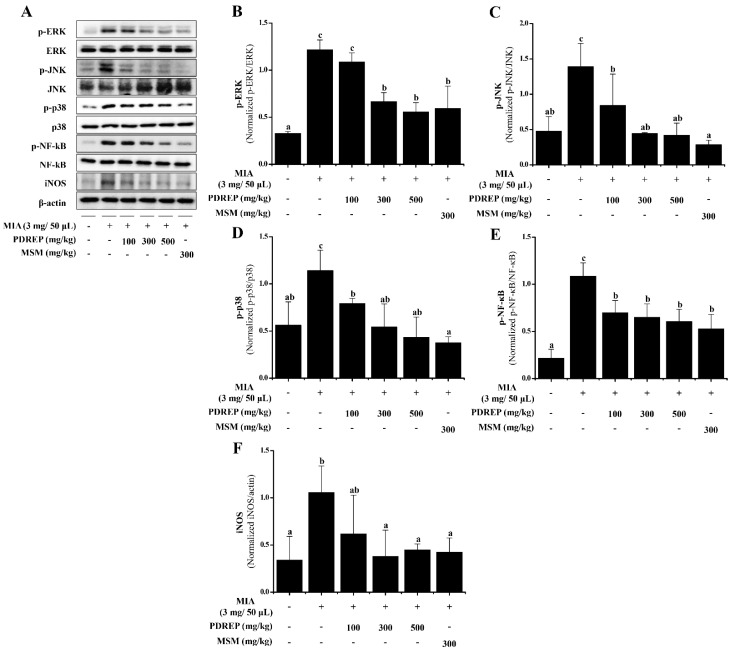
Effect of PDREP on MAPKs and NF-κB activation in knee joint cartilage tissues of MIA-induced OA rats. (**A**) Expression levels of ERK, p-ERK, p38, p-p38, JNK, p-JNK, NF-κB, p-NF-κB, and iNOS were examined by Western blot analysis. (**B**–**F**) Western blots were quantified using ImageJ software. Data are presented as mean ± SE (n = 3). Bars labeled with different superscript letters indicate *p* < 0.05.

To determine the effect of PDREP on the phosphorylation of ERK, JNK, p38, NF-κB, and the expression of iNOS in an MIA-induced OA rat model, cartilage was isolated. Proteins were extracted from the isolated cartilage, and the protein expression was confirmed by Western blotting (Figure 10A). In the control group treated only with MIA, the phosphorylation levels of ERK, JNK, p38, and NF-κB, as well as the expression level of iNOS, were significantly increased compared to the normal group. Conversely, the levels of p-JNK, p-p38, and p-NF-κB induced by MIA were significantly decreased in a dose-dependent manner with PDREP treatment (Figure 10C–E). For p-ERK and iNOS, significant decreases were observed in all experimental groups except for the group administered PDREP at 100 mg/kg compared to the control group (Figure 10B,F).

## 4. Discussion

Long-term drug treatment, physical therapy, surgery, and injection therapy are used to treat OA; however, as side effects and socioeconomic costs increase, research is being conducted on the use of natural products to prevent joint damage and reduce pain through inflammation control [18,21,22]. Among these natural products, MSM, which has been proven effective in alleviating OA, was selected as the positive control in this study [33]. *P. densiflora* is traditionally used to treat various conditions, including stroke, gastrointestinal disease, atherosclerosis, hypertension, diabetes, and neurological diseases [23,24,25]. Among them, *P. densiflora* root extract contains neutral polysaccharides, such as amylose and glucomannan, along with terpenoids and triterpenoids, and exhibits antioxidative, anti-inflammatory, antimalarial, antibacterial, and anticancer effects [23,24,25,26,27]. In this study, the components of PDREP were analyzed using UPLC–QTOF–MS, confirming the presence of a significant amount of azelaic acid (Supplementary Figure S1 and Table S1). Adelmidrol, a synthetic derivative of azelaic acid, is known to help suppress the inflammatory response associated with OA and arthritis [34]. However, there is no known research on whether PDREP has anti-inflammatory and joint improvement effects in OA. Therefore, in this study, we investigated the anti-inflammatory and joint improvement effects of PDREP using an IL-1β- and MIA-induced in vitro and in vivo OA model.

Inflammatory mediators (proteolytic enzymes, NO, etc.) and inflammatory cytokines (TNF-α, IL-1β, IL-6, IL-17, etc.) are reported to be deeply associated with the onset of OA [35,36]. An imbalance in the synthesis and degradation of the extracellular matrix (ECM) within the articular cartilage is considered the main cause of OA. The cohesive interaction between type II collagen and aggrecan, a proteoglycan found in cartilage, is essential for maintaining the integrity of a healthy cartilage matrix. MMPs degrade type II collagen and aggrecan in chondrocytes and inhibit their synthesis, causing cartilage degeneration and OA [37,38]. Among the MMPs, MMP-1, MMP-3, and MMP-13 were observed to have increased expression in OA, and have been reported to be closely related to the development of OA. In particular, increased production of MMPs by IL-1β is known to be one of the main mechanisms of OA induced by IL-1β [17]. Inflammatory cytokines, such as TNF-α, IL-1β, and IL-6, cause degeneration of articular cartilage and synovial inflammation, increase the death of chondrocytes, and inhibit the synthesis of ECM components in chondrocytes, causing OA [39,40]. Furthermore, it is known that patients with OA have higher levels of TNF-α, IL-1β, and IL-6 in their blood, synovial fluid, and cartilage tissue compared to healthy individuals [35]. Oxidative stress promotes synovial inflammation, chondrocyte apoptosis, inhibition of cartilage matrix synthesis, and associated intracellular signaling pathways related to OA [41]. Additionally, NO and COX-2 produced by iNOS are involved in the production of PGE2, which induces ECM degeneration and causes OA, and IL-1β is known to induce increases in iNOS and COX-2 [42]. In this study, the expression of MMPs (MMP-1, MMP-3, MMP-13), TNF-α, IL-6, NO, and PGE2 was significantly increased in chondrocytes treated with IL-1β. However, when treated with PDREP, the expression of MMPs (MMP-1, MMP-3, MMP-13), IL-6, and PGE2, which were increased by IL-1β, was significantly decreased. Additionally, type II collagen, which is known to decrease upon IL-1β treatment, increased the expression of Col2a1 upon PDREP treatment. These results suggest that PDREP may effectively improve OA by suppressing the inflammatory response and cartilage degradation of chondrocytes induced by IL-1β.

MAPKs (ERK, JNK, and p38) and NF-κB are known to be major signaling pathways involved in cartilage degeneration and OA progression [43,44,45]. Previous studies have reported that the activation of MAPKs signaling pathways regulates the expression of various genes involved in inflammation and induces ECM degeneration, leading to OA [43,46]. In addition, NF-κB is known to play the most important role in regulating the expression of inducible inflammatory mediators, cytokines, and MMPs in the pathogenesis of OA [44,47,48]. In particular, NF-κB is known to regulate the transcription of iNOS, COX-2, IL-1β, TNF-α, and IL-6, thereby controlling the generation of NO, PGE2, IL-1β, TNF-α, and IL-6 [49]. In this study, the results showed that the levels of ERK, JNK, p38, and NF-κB phosphorylation and iNOS expression, which were increased by IL-1β, were significantly decreased by PDREP treatment. These results suggest that PDREP exerts anti-inflammatory effects by inhibiting the activity of inflammation-related proteins involved in the inflammatory response and cartilage degradation in human SW1353 chondrocytes.

MIA induces OA by inhibiting the activity of glyceraldehyde-3-phosphate dehydrogenase in articular chondrocytes, leading to degeneration, joint damage, and functional impairment [50]. In a rat model, injection of MIA resulted in morphological changes in the synovium and surrounding tissues, accompanied by increased activity of osteoclasts and osteoblasts, resulting in articular cartilage loss and increased load on the subchondral bone [50,51]. OA is accompanied by severe swelling and pain in the inflamed area, causing the body to rely on the leg opposite to the inflamed area for support. This causes muscle loss due to movement disorders, and is known to have a specific walking pattern to adapt to pain [50,52]. Our results are consistent with those of previous studies [18,52,53], which showed that MIA-injected rats exhibited a significantly higher arthritis clinical index, decreased weight-bearing index, and reduced paw width and area than the normal group. In comparison with the MIA-induced control group, the PDREP and MSM groups showed a significantly suppressed arthritis clinical index, increased weight-bearing index, and increased paw width and area. These results show that PDREP reduced the OA clinical index in the OA rat model, thereby alleviating OA pain and improving gait patterns.

The meniscus, situated between the knee joints, plays a protective role. Previous studies have reported that MIA decreases joint volume and induces histological damage [18,50]. Therefore, micro-computed tomography (micro-CT) and histopathological examinations were performed to check for cartilage damage, a major symptom of OA. Micro-CT confirmed that the volume of cartilage between the femur and tibia increased in the PDREP and MSM groups (positive control) compared to the control group. Additionally, H&E staining revealed local subchondral damage in the high-concentration PDREP-treated group compared with that in the control group. However, cartilage loss was significantly reduced, leading to a recovery to a level similar to that observed in the positive control group. Safranin-O staining indicated that the PDREP and MSM groups showed relatively increased red areas compared to the control group, suggesting partial recovery of proteoglycan loss. Taken together, these results suggest that PDREP may effectively alleviate the clinical symptoms and joint tissue damage caused by OA.

Our in vivo study confirmed that PDREP exerts joint-improving and anti-inflammatory effects by regulating inflammatory cytokines and inflammation-related proteins, as demonstrated in our in vitro study. TNF-α plays a role in amplifying inflammation by activating cellular immunity and promoting the production of lower inflammatory mediators such as IL-2 and IL-6, which promote antibody production [53]. In addition, IL-1β produced by TNF-α has been reported to induce the production of inflammatory mediators such as PGE2 and NO in cartilage and synovial cells, as well as to stimulate the expression of MMPs (MMP-1, MMP-3, MMP-13), leading to cartilage degradation in OA [40,54,55]. In previous studies, it has been reported that MIA-induced OA animal models exhibit increased levels of pro-inflammatory cytokines (TNF-α, IL-6, IL-1β), inflammatory mediators (PGE2, NO), and MMPs (MMP-1, MMP-2, MMP-3, MMP-13) [6,15,18,56]. In addition, it was reported to increase the phosphorylation level of MAPKs (ERK, JNK, p38), which act as important regulators of the expression of inflammatory cytokines, and the level of NF-κB, which induces the synthesis of various inflammatory mediators [57,58,59,60]. Our results showed that the treatment of PDREP or MSM reduced the increased TNF-α, IL-6, PGE2, NO and MMPs (MMP-1, MMP-3, MMP-13) levels in an MIA-induced OA rat model. Additionally, when PDREP or the MSM was administered to a rat model of OA induced by MIA, the phosphorylation of ERK, JNK, and p38, which are MAPKs signaling pathways, and the expression levels of NF-κB phosphorylation and iNOS were significantly decreased. These results show that in the OA rat model, PDREP exhibited anti-inflammatory effects by reducing the levels of inflammatory cytokines and mediators and showed joint improvement effects by suppressing the destruction of synovial membranes and articular cartilage through decreased expression of MMPs. In addition, these results confirmed that PDREP exerts its effects by inhibiting the MAPKs and NF-κB signaling pathways. Taken together, we demonstrated that PDREP may exert anti-inflammatory and joint-improving effects by inhibiting the MAPKs/NF-κB signaling pathway and MMPs expression in in vitro and in vivo OA models induced by IL-1β and MIA.

This study aimed to evaluate the potential of PDREP as a health functional food or treatment strategy for improving OA. MSM, used as a positive control, is a healthy functional food commercially available as an OA supplement that has been recognized for its ability to relieve various types of pain and inflammation, including arthritis. In this study, PDREP demonstrated similar effects to MSM. This suggests that PDREP has qualities comparable to MSM as a functional food and holds promise as a supplement or complementary treatment for OA. One of the limitations is the lack of data on the plasma and metabolic stability of PDREP, which are crucial for determining its efficacy and safety in humans. Although our study demonstrated promising anti-inflammatory and joint-protective effects in preclinical models, further research is required to assess its pharmacokinetics, bioavailability, and long-term safety in clinical settings. Additionally, rigorous clinical trials are necessary to confirm the health functional food or treatment strategy of PDREP in OA patients. We plan to address these limitations in future studies to facilitate the clinical translation of PDREP as a potential therapeutic or supplementary product for OA.

## 5. Conclusions

In conclusion, our findings demonstrate the anti-inflammatory and joint-protective effects of PDREP in both in vitro and in vivo models of OA. PDREP reduced the expression of inflammatory cytokines, mediators, and MMPs in IL-1β-induced SW1353 chondrosarcoma cells and MIA-induced OA rat models, and it also decreased phosphorylation levels of MAPKs and NF-κB proteins. Furthermore, PDREP improved OA symptoms by reducing clinical signs and histological joint damage in OA rat models. These results suggest that PDREP may be a promising therapeutic strategy or functional product for OA, as it regulates inflammatory mediators and MMPs via the MAPKs/NF-κB signaling pathway, thereby reducing inflammation and preventing cartilage degradation.

## Figures and Tables

**Table 1:** Primer sequences used for qRT-PCR.

Species	Gene Names	Primer Sequences
Human	*TNF-α*	forward: 5′-GCCCAGGCAGTCAGATCATCT-3′ reverse: 5′-TTGAGGGTTTGCTACAACATGG-3′
Human	*IL6*	forward: 5′-ACTCACCTCTTCAGAACGAATTG-3′ reverse: 5′-CCATCTTTGGAAGGTTCAGGTTG-3′
Human	*MMP1*	forward: 5′-GATGGACCTGGAGGAAATCTTG-3′ reverse: 5′-TGAGCATCCCCTCCAATACC-3′
Human	*MMP3*	forward: 5′-GGTGTGGAGTTCCTGATGTTG-3′ reverse: 5′-AGCCTGGAGAATGTGAGTGG-3′
Human	*MMP13*	forward: 5′-TCAGGAAACCAGGTCTGGAG-3′ reverse: 5′-TGACGCGAACAATACGGTTA-3′
Human	*COL2A1*	forward: 5′-TGGTGATGATGGTGAAGCTG-3′ reverse: 5′-GAACCACTCTCACCCTTCAC-3′
Human	*GAPDH*	forward: 5′-AACAGCGACACCCACTCCTC-3′ reverse: 5′-GGAGGGGAGATTCAGTGTGGT-3′

## Data Availability

The data are available from the corresponding author upon reasonable requirement in the study.

## Supplementary Materials

The following supporting information can be downloaded at: https://www.mdpi.com/article/10.3390/nu16223882/s1, Figure S1: UPLC-QTOF-MS chromatogram of PDREP; Figure S2: UPLC-QTOF-MS chromatogram of PDREP; Figure S3: OARSI histological score; Table S1: Analysis of major metabolite components of PDREP by UPLC-QTOF-MS; Table S2: The catalog numbers and dilution ratios of the antibodies.
