# Supplementary material for: Pinus densiflora Root Extract Attenuates Osteoarthritis Progression by Inhibiting Inflammation and Cartilage Degradation in Interleukin-1β and Monosodium Iodoacetate-Induced Osteoarthritis Models
Source: Nutrients. 2024 Nov 14;16(22):3882. doi: 10.3390/nu16223882 (PMC11597245; doi:10.3390/nu16223882)
Supplement: Supplementary file 1 [file nutrients-16-03882-s001.zip › nutrients-3300689-supplementary.pdf]

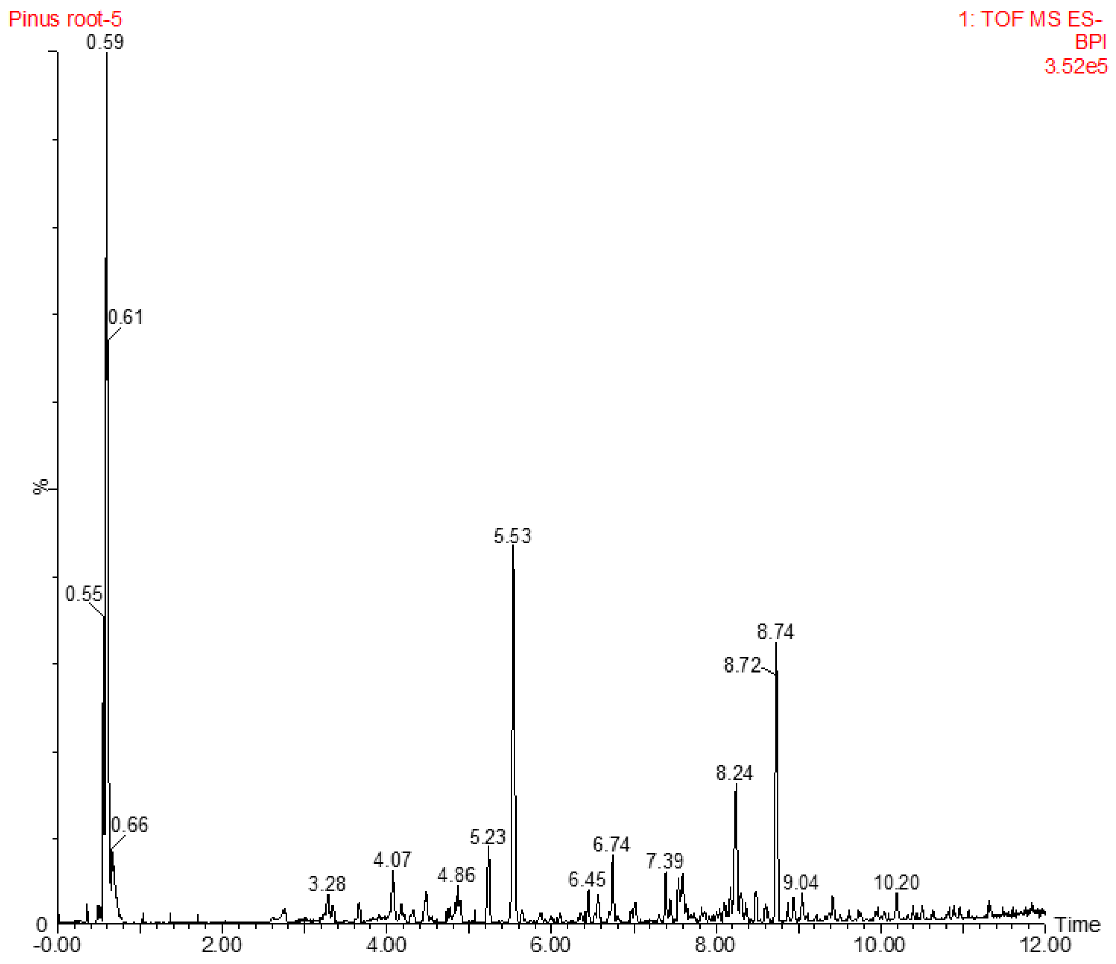

Figure S1. UPLC-QTOF-MS chromatogram of PDREP (retention time 12 min).

Table S1. Analysis of major metabolite components of PDREP by UPLC-QTOF-MS

No.	Tentative compound	RT (min)	Formula	Calculated MS [M-H]-	Observed MS [M-H]-	Mass error (ppm)	PDREP_Intensity
1	Lysine	0.52	$C_6H_{14}N_2O_2$	145.0977	145.0977	-0.03	349.02
2	Histidine	0.52	$C_6H_9N_3O_2$	154.0617	154.0617	-0.02	409.70
3	Arginine	0.52	$C_6H_{14}N_4O_2$	173.1039	173.1034	-0.48	123.64
4	Asparagine	0.56	$C_4H_8N_2O_3$	131.0457	131.0414	-4.30	585.13
5	Quinic acid	0.60	$C_7H_{12}O_6$	191.0556	191.0558	0.17	8137.93
6	Fumaric acid	0.63	$C_4H_4O_4$	115.0031	115.0036	0.51	134.22
7	Isocitric acid	0.63	$C_6H_8O_7$	191.0192	191.0193	0.09	3431.54
8	Tyrosine	0.83	$C_9H_{11}NO_3$	180.0661	180.0661	0.01	133.30
9	Phenylalanine	1.45	$C_9H_{11}NO_2$	164.0712	164.0713	0.14	1386.38
10	Tryptophan	2.50	$C_{11}H_{12}N_2O_2$	203.0821	203.0818	-0.29	341.88
11	4-hydroxybenzoic acid	2.75	$C_7H_6O_3$	137.0239	137.0239	-0.04	2123.99
12	Vanillic acid	3.36	$C_8H_8O_4$	167.0344	167.0339	-0.50	115.08
13	Pimelic acid	3.39	$C_7H_{12}O_4$	159.0657	159.0654	-0.27	310.46
14	p-Coumaric acid	4.19	$C_9H_8O_3$	163.0395	163.0397	0.23	120.61
15	Taxifolin 3-glucoside	4.36	$C_{21}H_{22}O_{12}$	465.1033	465.1033	0.03	598.80
16	Lariciresinol 4-O-glucoside	4.55	$C_{26}H_{34}O_{11}$	521.2023	521.2037	1.45	291.26
17	trans-ferulic acid	4.60	$C_{10}H_{10}O_4$	193.0501	193.0499	-0.23	237.12
18	Quercetin-3-D-galactoside(Hyperoside)	4.60	$C_{21}H_{20}O_{12}$	463.0877	463.0876	-0.07	98.31
19	Quercetin 3-o-glucoside(isoquercitrin)	4.67	$C_{21}H_{20}O_{12}$	463.0877	463.0870	-0.73	336.13
20	Kaempferol-3-O-glucoside	4.78	$C_{21}H_{20}O_{11}$	447.0927	447.0938	1.07	459.35
21	Laricitrin 3-O-glucoside	4.86	$C_{22}H_{22}O_{13}$	493.0982	493.0987	0.46	7727.68
22	Azelaic acid	5.53	$C_9H_{16}O_4$	187.097	187.0976	0.60	27384.12

23	2-Hydroxycinnamic acid	5.60	$C_9H_8O_3$	163.0395	163.0388	-0.67	191.25
24	Piceid	5.65	$C_{20}H_{22}O_8$	389.1236	389.1225	-1.10	192.16
25	Myricetin 3-O-rhamnoside	5.65	$C_{21}H_{20}O_{12}$	463.0877	463.0879	0.19	1403.39
26	Secoisolariciresinol	5.74	$C_{20}H_{26}O_6$	361.1651	361.1651	0.03	853.50
27	Abscicic acid	6.45	$C_{15}H_{20}O_4$	263.1283	263.1306	2.32	115.77
28	Sebacic acid	6.56	$C_{10}H_{18}O_4$	201.1127	201.1123	-0.39	3511.35
29	Pinoresinol	7.01	$C_{20}H_{22}O_6$	357.1338	357.1322	-1.57	290.86
30	Matairesinol	7.44	$C_{20}H_{22}O_6$	357.1338	357.1338	0.04	3254.79
31	Carnosol	8.74	$C_{20}H_{26}O_4$	329.1753	329.1758	0.54	21380.11
32	Carnosic acid	8.87	$C_{20}H_{28}O_4$	331.1909	331.1907	-0.18	2288.96

UPLC-QTOF-MS analysis of PDREP

Metabolite components for PDREP were analyzed in ESI negative mode by UPLC-QTOF MS. Most peaks were detected within 12 minutes and are shown in Fig. S1. Through PDREP metabolite profiling, 32 flavonoids, phenolic compounds, amino acids, and organic acids were identified (Table S1). Table S1 shows the molecular formula and substance name estimated from the information obtained for each peak through the metabolite profile of PDREP. PDREP: *Pinus densiflora* root extract powder

Table S2. The catalog numbers and dilution ratios of the antibodies

Antibody	Manufacturer	Cat. No.	Dilution times (ratio)
ERK	Cell Signaling	#9102S	1 : 2000
p-ERK	Cell Signaling	#9101S	1 : 2000
JNK	Cell Signaling	#9252S	1 : 2000
p-JNK	Cell Signaling	#4671S	1 : 2000
p38	Cell Signaling	#9212S	1 : 2000
p-p38	Cell Signaling	#4511S	1 : 2000
NF-kB	Cell Signaling	#8242S	1 : 2000
p-NF-kB	Cell Signaling	#3033S	1 : 2000
iNOS	Abcam	ab178945	1 : 2000
β-actin	Santa Cruz Biotechnology	sc-47778	1 : 4000

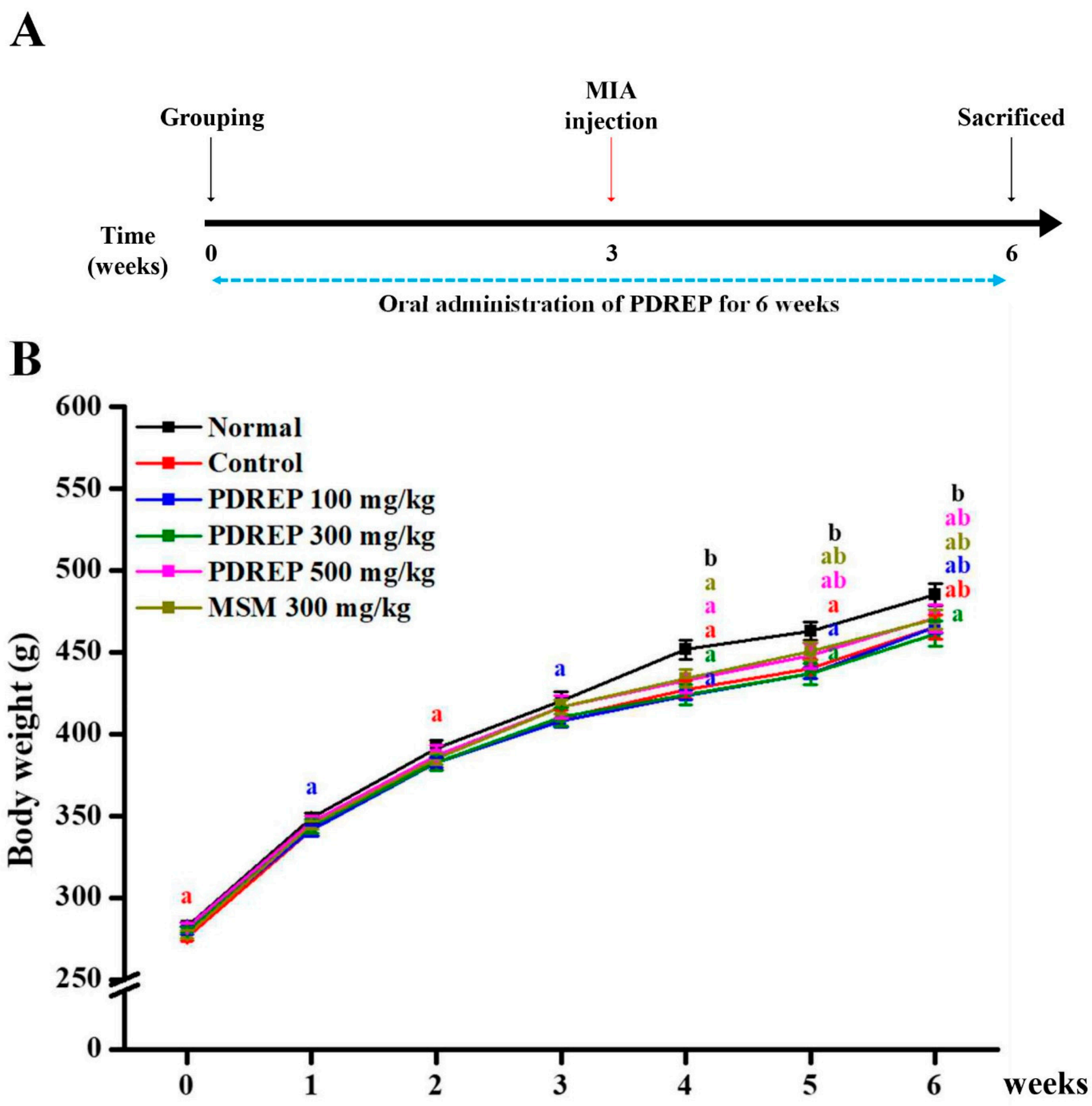

Figure S2. Effects of PDREP on body weight change in MIA-induced OA rats. (A) Treatment schedule. (B) The body weight of rats was measured once per week for 6 weeks. The data are expressed as the mean ± SE (n = 10), and values in the row with different superscript letters are significantly different, $p < 0.05$. PDREP: *Pinus densiflora* root extract powder; MSM: Methyl sulfonyl methane

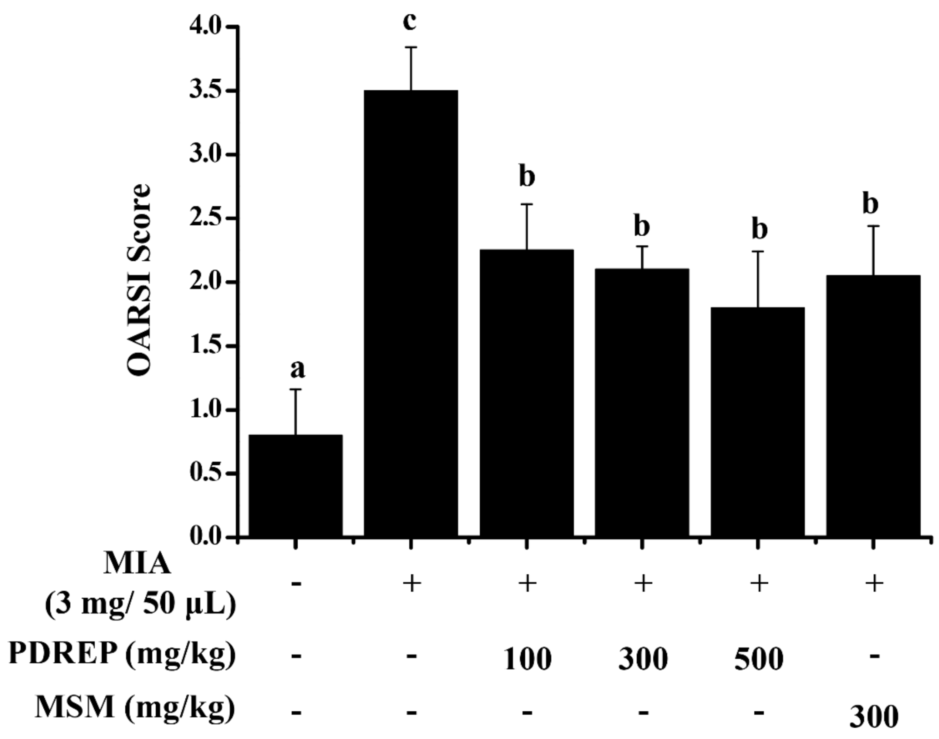

Figure S3. OARSI histological score. OARSI was evaluated. The data are expressed as the mean ± SE (n = 5). Statistical analysis indicated that the differences were significant, between the normal group and control group, as well as between PDREP or MSM groups and control group. Bars labeled with different superscript letters indicate p values < 0.05.
